# Teaching the Heart Before It Breaks: The Scientific Case for Primordial Prevention in Childhood

**DOI:** 10.1016/j.jacadv.2026.103232

**Published:** 2026-09-11

**Authors:** Ramzi Ibrahim

**Affiliations:** Department of Cardiovascular Medicine, Mayo Clinic, Phoenix, Arizona, USA

**Keywords:** cardiovascular health, population, prevention

I arrived at the inaugural Fuster Prevention Forum expecting to be inspired, and I was. But I left with something more useful than inspiration: a question I could not answer. If atherosclerosis begins in childhood, why does a profession that knows this so well still spend most of its energy repairing hearts that have already failed? The premise behind the Forum, and behind a growing body of evidence, is that the intervention with the greatest leverage over a person's cardiovascular future may occur decades before that person ever meets a cardiologist.

The reason begins with a fact that has quietly transitioned into consensus. Atherosclerosis is not a disease of old age that we happen to detect late; it develops early and advances silently for decades before the first symptom. The PESA-CNIC-Santander study made this visible, documenting widespread subclinical atherosclerosis among asymptomatic adults still in their forties, with younger individuals proving especially vulnerable to the arterial injury caused by elevated cholesterol and blood pressure.^1^ What makes the finding sobering is not that high-risk adults harbor hidden disease, but that risk-factor levels long considered unremarkable still leave a measurable atherosclerotic footprint in the young and track toward events later in life.^2^ Disease, in other words, is already underway in people we would never think to treat.

If the process starts this early, the logic of when to intervene shifts with it. Here a distinction matters. Primary prevention modifies risk factors once they have appeared; primordial prevention aims to keep them from appearing at all.^3^ The 2 are not rivals but partners along a single continuum, and neither suffices alone. A clinician managing an adult's newly elevated blood pressure is practicing the former; a community keeping a child's blood pressure from ever rising is practicing the latter. Because the behaviors that drive cardiovascular risk are largely established in childhood, that early window offers a rare and cost-effective chance to impact a lifelong trajectory before it sets.^3^

Nowhere is the arithmetic of early action clearer than with low-density lipoprotein cholesterol (LDL-C). What injures the artery wall is not a single cholesterol value but the total atherogenic burden carried over time, the height of the elevation multiplied by its duration. Ference et al^4^ have framed this as a lifetime accrual: apolipoprotein B-containing particles become trapped in the arterial wall, plaque grows as the trapping continues, and sustaining a lower LDL-C postpones the age at which mature, rupture-prone plaque appears, lowering lifetime risk in proportion. Braunwald^5^ captured the idea in an image every cardiologist understands, describing the accumulating burden in “cholesterol-years,” much as we count pack-years of smoking, and locating the threshold for clinical events at roughly 7 LDL-C gram-years.

When that burden is acquired appears to matter as much as how much of it accumulates. In the CARDIA cohort, Domanski et al^6^ found that elevated LDL-C between the ages of 18 and 40 predicted events after age 55 more strongly than comparable exposure encountered later, suggesting that risk banked early is not fully refundable by treatment begun late. Mendelian randomization points the same direction, with lifelong genetically lower LDL-C conferring protection that far exceeds what an identical reduction achieves when started in middle age^4^ (Figure 1). This is the quantitative heart of Braunwald's provocation that one might reasonably aspire to reach 100 before developing clinical coronary disease.^5^

**Figure 1:**
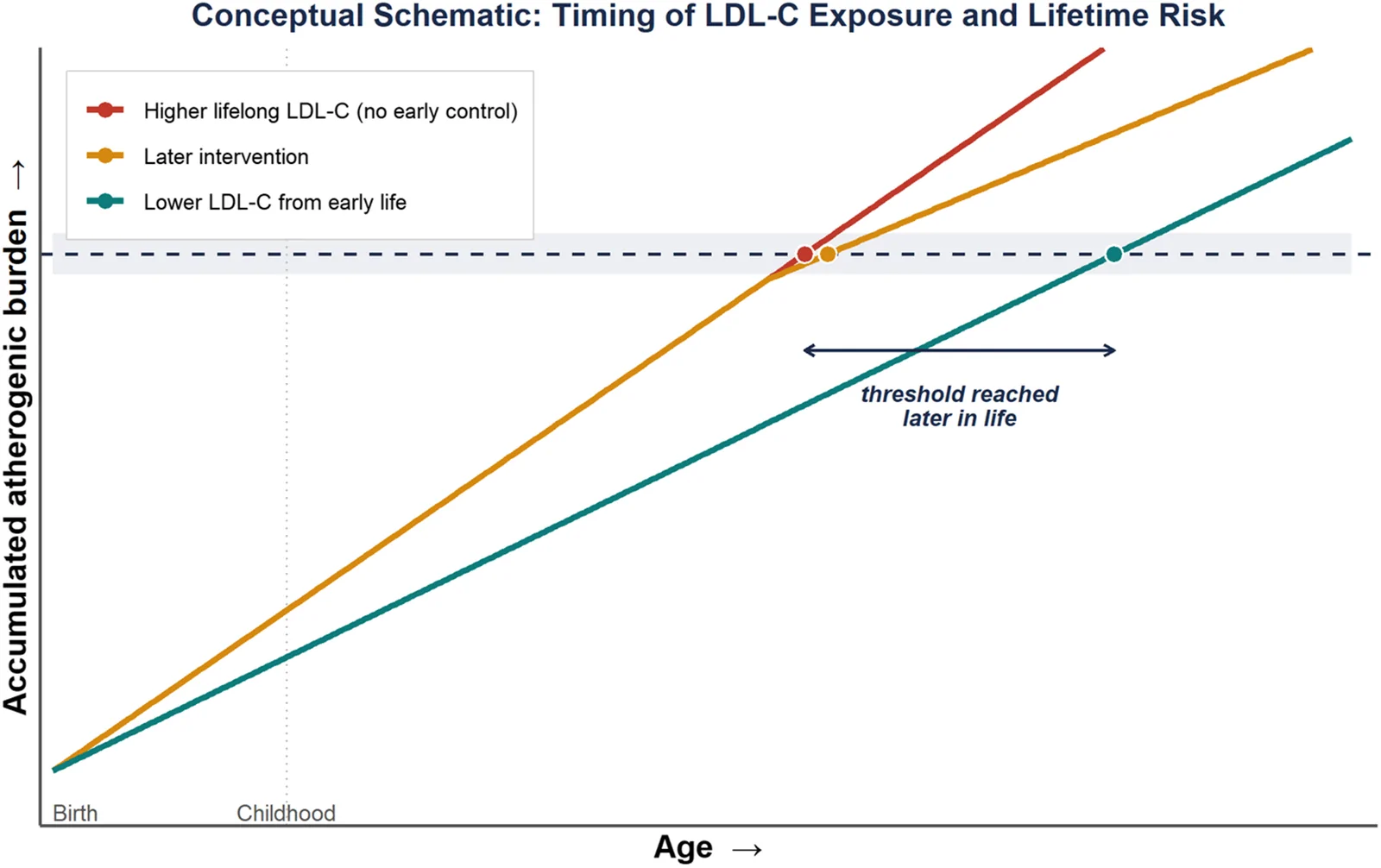
Conceptual Schematic: Timing of LDL-C Exposure and Lifetime Cardiovascular Risk Conceptual schematic of the relationship between age, the timing of LDL-C exposure, and lifetime cardiovascular risk. ∗The curves are illustrative and not to scale. The dashed line denotes a conceptual threshold beyond which clinical events become likely, corresponding to the “cholesterol-years” burden described by Dr. Braunwald. The schematic conveys a single qualitative point drawn from references 4 through 6. The figure is not derived from patient-level data and is intended only to illustrate this concept. LDL-C = low-density lipoprotein cholesterol.

None of this diminishes the treatments we use in adulthood; it reframes them as the later, costlier half of a job that could have started sooner. The American Heart Association's Life's Essential 8 reflects that reframing, extending its definition of cardiovascular health across the full life course from age 2 onward.^7^ The framework's own authors note that the dividend from optimizing these metrics, spanning diet, physical activity, nicotine exposure, sleep, weight, lipids, glucose, and blood pressure, is largest in the young, who have the most years of arterial health left to protect.^7^ Higher scores track with lower long-term cardiovascular and all-cause mortality, and favorable health in childhood tends to persist into adulthood.^8^

Recognizing this is easier than acting on it, because the determinants of early cardiovascular health lie mostly outside the clinic. Diet, movement, and emotional regulation are learned, absorbed in homes, schools, and neighborhoods during the years when habits form most durably. This is the gap that the prevention research associated with Valentin Fuster was designed to close. The SI! Program (Salud Integral), refined over a decade across Colombia, Spain, and the United States, showed in cluster-randomized trials that structured, school-based education can improve knowledge, attitudes, and habits in children as young as 3, with the strongest effects among those who remained in the program longest and measurable reductions in adiposity after sustained exposure.^9^ Extending the model into preschools serving predominantly Hispanic and African-American communities placed it precisely where cardiovascular risk tends to run highest.^9^ Whether such early behavioral gains ultimately translate into fewer hard cardiovascular events is a question these programs are still maturing to answer; the randomized PRECAD (Prevent Coronary Artery Disease) trial now tests a complementary piece, asking whether aggressive risk-factor control in otherwise healthy adults under 40 can alter the atherosclerotic course itself.^2^

What unites these efforts is a quiet expansion of the cardiologist's role, from a clinician who repairs to one who also teaches and organizes. That expansion has implications worth stating plainly, if tentatively. We have built extraordinary capacity to treat established disease while devoting only a small fraction of health spending to preventing it.^10^ If risk accumulates from childhood, if the timing of LDL-C exposure shapes lifetime events, and if the habits underlying cardiovascular health are set early, then it is at least reasonable to ask whether our patterns of training, workforce development, and investment should tilt further upstream than they currently do. That need not mean dismantling what works; it might mean treating a third-grade classroom as a legitimate extension of cardiovascular practice, and preparing clinicians to work there.

The inaugural Fuster Prevention Forum is one attempt to build that capacity, equipping clinicians to carry the science of early prevention back into their own communities. It would be an overstatement to say the case for childhood prevention is closed. Much of the strongest evidence still comes from observational cohorts, genetic studies, and intermediate outcomes rather than randomized trials with hard endpoints, and that gap deserves honesty rather than enthusiasm. But the direction of the evidence is consistent, the biology is coherent, and the potential return is measured in decades of life. That seems reason enough to begin taking the early years seriously and to try to reach the heart before it breaks.

## Funding support and author disclosures

The author participated in the inaugural 2026 Fuster Prevention Forum cohort. The author has reported that they have no relationships relevant to the contents of this paper to disclose.
